# Unlocking the Role of OCT4 in Cancer Lineage Plasticity: A Cross-Cancer Perspective with an Emphasis on Prostate Cancer

**DOI:** 10.3390/biomedicines13071642

**Published:** 2025-07-04

**Authors:** Mohammad Esfini Farahani, Yanquan Zhang, Amos Olalekan Akinyemi, Fatemeh Seilani, Md Rakibul Alam, Xiaoqi Liu

**Affiliations:** 1 Department of Toxicology and Cancer Biology, Collage of Medicine, University of Kentucky, Lexington, KY 40536, USA; mesfarahani@uky.edu (M.E.F.); yanquan.zhang@uky.edu (Y.Z.); amos.olalekan7@uky.edu (A.O.A.); fatemeh.seilani@uky.edu (F.S.); rakibul.alam@uky.edu (M.R.A.); 2 Markey Cancer Center, University of Kentucky, Lexington, KY 40536, USA

**Keywords:** octamer-binding transcription factor 4, prostate cancer stem cells, androgen deprivation therapy, castration-resistant prostate cancer, neuroendocrine prostate cancer

## Abstract

Prostate cancer (PCa) is a highly heterogeneous disease, with castration-resistant prostate cancer (CRPC) and neuroendocrine prostate cancer (NEPC) representing its most aggressive and therapy-resistant forms. Emerging evidence indicates that lineage plasticity—driven by key transcription factors such as Octamer Binding Factor 4 (OCT4)—plays a crucial role in therapeutic resistance and disease progression. OCT4, in coordination with SOX2 and NANOG, acts as a master regulator of stemness and is frequently upregulated in prostate cancer stem cells (PCSCs). This upregulation contributes to tumor initiation, metastasis, and resistance to both androgen deprivation therapy (ADT) and chemotherapy. In this review, we explore the role of OCT4 in mediating lineage plasticity in prostate cancer, with particular emphasis on its involvement in treatment resistance and neuroendocrine differentiation. We also examine therapeutic strategies aimed at targeting OCT4 directly, such as microRNA-mediated suppression, small-molecule inhibitors, and suicide gene therapy, as well as indirect approaches that modulate OCT4 expression via FGFR and NF-κB signaling pathways. While these strategies offer promising avenues, challenges such as adaptive resistance and the intricate signaling networks within PCSCs remain significant hurdles. A deeper understanding of the molecular mechanisms underlying OCT4-driven plasticity may pave the way for novel therapeutic approaches and improved outcomes in advanced prostate cancer.

## 1. Introduction

PCa remains one of the most frequently diagnosed malignancies in men, ranking among the leading causes of cancer-related mortality worldwide [1]. The disease predominantly affects aging populations, with incidence rates rising significantly in men over 50 years old [2]. While early-stage PCa can often be effectively managed through localized interventions, such as surgery and radiation therapy, advanced cases present a major therapeutic challenge. Androgen deprivation therapy (ADT) has long been the cornerstone of treatment for metastatic prostate cancer [3,4]; however, most patients eventually develop castration-resistant prostate cancer (CRPC), an aggressive stage of the disease characterized by continued tumor growth despite androgen suppression [5,6]. A subset of CRPC cases undergoes further progression to neuroendocrine prostate cancer (NEPC), a highly aggressive and therapy-resistant variant with limited treatment options [7,8]. The molecular mechanisms driving this transition are complex and remain an area of intense research.

A key emerging concept in PCa progression is lineage plasticity, which refers to the ability of cancer cells to alter their differentiation state and adopt new cellular identities in response to therapeutic pressures [9]. This phenomenon has been recognized as a major driver of resistance to the androgen receptor (AR)-targeted therapies, such as enzalutamide and abiraterone, which are designed to block AR signaling and inhibit tumor growth [10,11]. However, some PCa cells evade these therapies by undergoing a lineage switch, losing their dependence on AR and transitioning into alternative states, including neuroendocrine-like or stem-like phenotypes. This process allows tumor cells to survive and proliferate even in the absence of androgen signaling, making them highly resistant to standard treatments [12,13].

Several molecular regulators contribute to lineage plasticity in PCa, including transcription factors, epigenetic modifiers, and oncogenic drivers. Pluripotency-associated factors such as OCT4 (POU5F1), SOX2, and NANOG are frequently implicated in dedifferentiation and cancer stem cell (CSC) maintenance, allowing tumor cells to regain developmental plasticity [14,15]. Concurrently, the emergence of neuroendocrine features is marked by the upregulation of synaptophysin (SYP), neuron-specific enolase (NSE/ENO2), and chromogranin A (CHGA), which are commonly used as biomarkers for NEPC. Additionally, epigenetic regulators such as enhancer of zeste homolog 2 (EZH2) and lysine-specific demethylase 1 (LSD1) have been shown to facilitate lineage reprogramming by altering chromatin states and repressing luminal differentiation programs. Oncogenic drivers, including Aurora kinase A (AURKA) and MYCN, further promote the transition from prostate adenocarcinoma to NEPC by stabilizing neuroendocrine transcriptional regulators and disrupting epithelial lineage commitment [16].

OCT4, a transcription factor best known for its essential role in maintaining pluripotency in embryonic stem cells, is among the key regulators of lineage plasticity. OCT4 functions as a master regulator of self-renewal and prevents differentiation, ensuring that stem cells retain their ability to give rise to multiple lineages [17,18]. While its developmental function is well established, accumulating evidence suggests that OCT4 is aberrantly re-expressed in various cancers, including PCa, where it contributes to tumor initiation, cancer stemness, therapy resistance, and disease progression. By sustaining CSC populations, OCT4 enables tumor cells to resist therapy, remain dormant, and later re-emerge as highly aggressive, treatment-resistant clones [19,20].

In prostate cancer, OCT4′s influence extends beyond cancer stem cell maintenance: its overexpression has been associated with increased tumor aggressiveness, enhanced metastatic potential, and resistance to both ADT and chemotherapy [21,22]. One mechanism by which OCT4 contributes to disease progression is through its regulation of epithelial–mesenchymal transition (EMT), a biological process that grants epithelial cells mesenchymal-like properties, including increased motility and invasiveness. EMT is a key driver of metastasis in several cancers, including PCa, and OCT4 has been shown to activate EMT-associated gene expression programs [23,24].

A particularly intriguing aspect of OCT4′s function in PCa is its interaction with the androgen receptor (AR)-signaling pathway, which is the primary driver of PCa growth. In early stages, tumors are highly dependent on AR signaling, rendering them sensitive to AR-targeted therapies. However, as the disease advances, cancer cells often develop mechanisms to bypass AR dependency. Emerging evidence shows that OCT4 plays a role in this transition by driving the loss of AR signaling and promoting androgen-independent growth, thereby contributing to therapy resistance [25]. More recently, studies have highlighted OCT4′s involvement in the development of treatment-induced NEPC, further underscoring its role in lineage plasticity and disease progression [15,26,27].

Given its multifaceted role in prostate cancer biology, OCT4 has emerged as both a potential biomarker and a therapeutic target [28,29]. Elevated OCT4 expression has been associated with poor clinical outcomes, including increased metastatic potential and reduced overall survival [30]. As such, OCT4 could serve as a valuable biomarker for identifying patients at a high risk of aggressive disease and therapy resistance [31,32]. Additionally, targeting OCT4 directly—or disrupting its associated pathways—represents a promising strategy to overcome therapy resistance [33]. Several experimental approaches, including small-molecule inhibitors, RNA-based therapies, and CRISPR-mediated gene editing, are currently under exploration [34,35,36].

This review aims to provide a comprehensive examination of OCT4′s role in PCa, with a particular focus on its contributions to lineage plasticity, disease progression, and therapy resistance. We will explore the molecular mechanisms by which OCT4 regulates cancer stemness, EMT, and AR independence, as well as its interactions with key oncogenic pathways. Additionally, we will discuss the potential of OCT4 as a biomarker for aggressive PCa and evaluate emerging therapeutic strategies aimed at targeting OCT4. By gaining a deeper understanding of OCT4′s role in PCa, we can uncover new opportunities for improving the diagnosis, prognosis, and treatment of this challenging disease.

## 2. OCT4 in Stemness and Plasticity

### 2.1. OCT4′s Function in Embryonic Stem Cells and Normal Tissue Development

OCT4, also known as POU5F1, is a pivotal transcription factor in embryonic stem cells (ESCs), essential for maintaining pluripotency and self-renewal [37,38]. It regulates genes critical for cell fate determination, such as NANOG and SOX2. In normal development, the OCT4 expression is tightly controlled and diminishes as cells differentiate [39]. However, aberrant reactivation of OCT4 in adult tissues has been linked to tumorigenesis and therapy resistance in various cancers, including PCa [15,20,24,31,40,41,42,43,44].

OCT4′s functional versatility is partly attributed to its interaction with multiple epigenetic regulators. A recent study by Ding et al. [45] identified several key binding partners of OCT4 involved in chromatin remodeling and gene regulation, including Supt16h, Zmym2, Ring1B/Rnf2, Msh2/6, Ash2l, Kif11, and Ppp1cc—all of which contribute to stem cell maintenance and somatic cell reprogramming. Additionally, OCT4 cooperates with other transcription factors, particularly SOX2, to recognize and bind to specific DNA elements, forming regulatory complexes that govern gene expression programs in both pluripotent and cancer cells [46].

Beyond transcriptional regulation, OCT4 is also subject to post-translational modifications (PTMs) that fine-tune its stability and activity. Notably, ubiquitination and phosphorylation have been shown to influence OCT4 protein turnover and function, providing another layer of control over its biological effects [47,48,49].

### 2.2. OCT4 in Cancer Stem-like Cells and Lineage Plasticity

Cancer stem-like cells (CSCs) represent a subpopulation of tumor cells with enhanced self-renewal capacity, plasticity, and resistance to therapy. These CSCs are believed to contribute to tumor initiation, progression, and metastasis. Therefore, they are considered cancer therapeutic targets [50,51]. OCT4 is a major regulator of CSC biology. It contributes to neovasculogenesis in vivo and sustains CSC populations by maintaining their undifferentiated state and promoting a stem-like transcriptional program [52].

In PCa, elevated OCT4 expression has been consistently linked with advanced and therapy-resistant disease states, including CRPC and NEPC [15], facilitating the transition from androgen-dependent adenocarcinoma to more aggressive, therapy-resistant forms. Consistently, a research group found that NEPC exhibits molecular features reminiscent of stem cells, further reinforcing the link between OCT4-driven stemness and lineage plasticity [53]. This plasticity enables cancer cells to evade androgen receptor (AR)-signaling dependence, contributing to the resistance against treatments like enzalutamide and abiraterone [10,54].

### 2.3. OCT4-Associated Signaling Pathways in CSC and Plasticity

#### 2.3.1. Wnt/β-Catenin

The Wnt/β-catenin signaling pathway plays a critical role in regulating embryonic development, stem cell maintenance, and tissue homeostasis. However, dysregulation of this pathway is frequently implicated in tumorigenesis. In several cancers—including colorectal, breast, liver, and prostate cancer—mutations in key regulatory components, such as APC or β-catenin (CTNNB1), lead to aberrant pathway activation. Constitutive Wnt/β-catenin signaling results in uncontrolled cellular proliferation, enhanced tumor aggressiveness, and increased resistance to therapy. In the context of prostate cancer, aberrant activation of this pathway has been linked to disease progression, therapy resistance, and maintenance of cancer stem-like cells, potentially through interactions with pluripotency factors like OCT4 [55,56,57].

#### 2.3.2. TGF-β

The TGF-β (transforming growth factor-beta) superfamily comprises a broad group of structurally related growth factors that regulate essential cellular processes, including development, proliferation, apoptosis, metabolism, and differentiation. In the context of cancer, TGF-β signaling exhibits a dual role, acting as a tumor suppressor in the early stages of the disease and as a tumor promoter in advanced cancers. During the initial stages of tumor development, TGF-β restricts the proliferation of epithelial and immune cells, preserves genomic stability, and suppresses mitogenic signaling, thereby functioning as a tumor suppressor. However, as cancer progresses, genetic and epigenetic alterations can subvert this pathway, shifting TGF-β’s role from growth inhibition to tumor promotion. In later stages, TGF-β signaling facilitates tumor cell proliferation, invasion, epithelial–mesenchymal transition (EMT), and metastasis, contributing to disease progression and therapeutic resistance [58,59].

#### 2.3.3. PI3K/AKT/mTOR

The PI3K/AKT/mTOR signaling pathway is a central regulator of cell survival, growth, metabolism, and proliferation, playing a crucial role in normal physiology and cancer progression. Under normal conditions, this pathway is activated by growth factors that bind to receptor tyrosine kinases (RTKs), leading to the phosphorylation of PI3K, which, in turn, activates AKT. Once activated, AKT promotes cell survival by inhibiting pro-apoptotic proteins and stimulating cell cycle progression. Further downstream, mTOR acts as a key effector, driving protein synthesis, metabolic reprogramming, and tumor growth. In cancer, aberrant activation of this pathway—often due to genetic mutations or loss of regulatory control—leads to uncontrolled proliferation, resistance to apoptosis, and enhanced metastatic potential. Moreover, PI3K/AKT/mTOR hyperactivation is associated with therapy resistance in various cancers, including parathyroid carcinoma, prostate, breast, colorectal, and lung cancer. Given its pivotal role in tumor progression, this pathway has emerged as a critical therapeutic target. Several inhibitors, such as PI3K inhibitors (Buparlisib, Alpelisib), AKT inhibitors (Capivasertib, Ipatasertib), and mTOR inhibitors (Everolimus, Temsirolimus), have been developed to block different components of the cascade. Additionally, combination therapies targeting PI3K/AKT/mTOR alongside conventional treatments, such as chemotherapy or immune checkpoint inhibitors, are being explored to improve patient outcomes [60,61,62].

#### 2.3.4. Notch

The Notch signaling pathway is a highly conserved cell communication system that regulates critical cellular processes, including proliferation, differentiation, and apoptosis. Dysregulation of Notch signaling has been implicated in the progression of various cancers, largely due to its role in maintaining (CSCs) a subpopulation responsible for tumor initiation, metastasis, therapy resistance, and disease recurrence.

In prostate cancer and other solid tumors, aberrant Notch activity supports CSC survival and enhances tumor aggressiveness. Its interaction with key pluripotency regulators such as OCT4 may further reinforce stem-like properties and contribute to lineage plasticity, particularly under therapeutic pressure.

Given the central role of CSCs in treatment failure and tumor relapses, targeting Notch signaling has emerged as a promising strategy to eliminate these therapy-resistant populations. However, the context-dependent effects of Notch—acting as either an oncogene or tumor suppressor depending on the tissue type and disease stage—pose significant challenges for therapeutic targeting. Ongoing efforts aim to develop selective modulators of Notch activity that can effectively disrupt its oncogenic functions while minimizing unintended effects on normal tissues [63,64,65].

#### 2.3.5. JAK1-STAT3

The JAK1-STAT3 signaling pathway is a key regulator of oncogenic processes, including cell proliferation, survival, and immune evasion. The aberrant and sustained activation of this pathway has been widely observed in multiple cancers and is particularly associated with the maintenance of cancer stem cells (CSCs), which drive tumor initiation, metastasis, and therapeutic resistance. STAT3 functions as a transcription factor that, when persistently activated, promotes an inflammatory tumor microenvironment, enhances stemness-related gene expression, and supports immune suppression. This immune evasion enables tumor cells to persist and expand despite host immune responses and therapeutic interventions.

In prostate cancer, chronic activation of the JAK1-STAT3 axis has been linked to lineage plasticity and disease progression. Its interaction with stemness regulators like OCT4 may further enhance the CSC properties, reinforcing therapy resistance and enabling phenotypic switching, such as neuroendocrine differentiation under androgen-deprived conditions [66,67,68].

#### 2.3.6. ERK-MAPK

The ERK-MAPK signaling pathway is a critical regulator of cell proliferation, survival, differentiation, and apoptosis. Its dysregulation has been widely implicated in cancer progression, contributing to increased tumor aggressiveness, therapy resistance, and metastasis. This pathway is often hyperactivated in cancer due to mutations in upstream regulators such as receptor tyrosine kinases or RAS proteins. Notably, cancer stem cells (CSCs) exploit ERK-MAPK signaling to maintain their self-renewal capacity and resistance to conventional therapies. Given its central role in tumor biology, targeting ERKMAPK signaling has emerged as a promising therapeutic strategy to inhibit cancer progression and sensitize tumors to treatment [69,70,71].

Understanding the interplay between OCT4 and these signaling pathways provides insights into the mechanisms underlying cancer stemness and plasticity, offering potential therapeutic targets for combating aggressive and treatment-resistant cancers.

**Figure 1:**
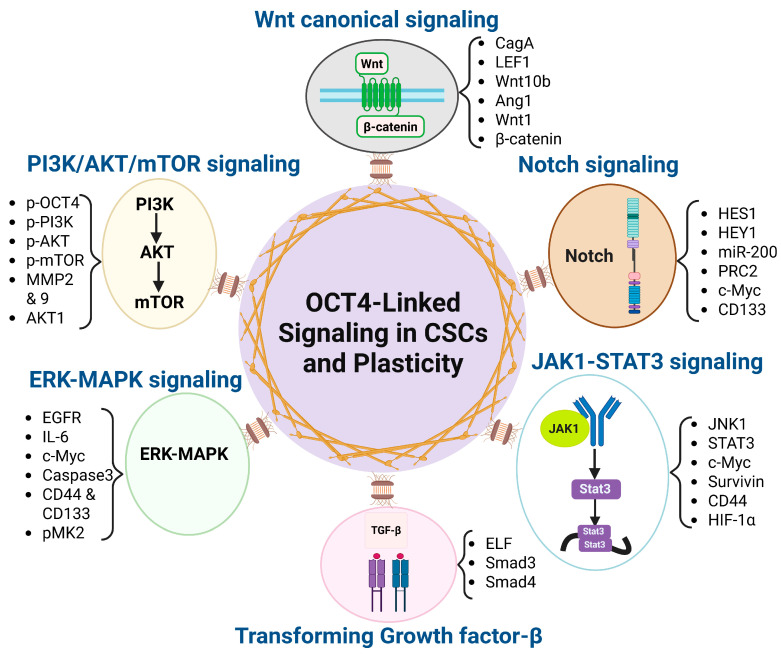
OCT4-associated signaling pathways driving cancer stemness and plasticity. This diagram illustrates the central role of OCT4 in regulating cancer stem cell (CSC) properties and phenotypic plasticity through its interaction with six key signaling pathways (Wnt/β-catenin, TGF-β, PI3K/AKT/mTOR, Notch, JAK1-STAT3, and ERK-MAPK) and their correlated down-stream effectors. These pathways contribute to the maintenance of stemness, promotion of epithelial–mesenchymal transition (EMT), therapy resistance, and tumor progression.

The following table (Table 1) and Figure 1 summarize the role of OCT4 in these signaling pathways:

## 3. OCT4 in Prostate Cancer Progression and Lineage Plasticity

As discussed, OCT4 has been extensively studied for its role in maintaining pluripotency in embryonic stem cells. However, its aberrant expression in cancerous tissues—particularly in solid tumors—has been linked to tumorigenesis and aggressive disease progression, making it a potential target for eliminating cancer stem cells (CSCs) in various malignancies, including prostate cancer (PCa) [107]. In PCa, OCT4 is upregulated in tumor cells compared to normal prostate epithelium, suggesting its involvement in prostate cancer development.

### 3.1. OCT4 as a Driver of Prostate Cancer Initiation

**Figure 2:**
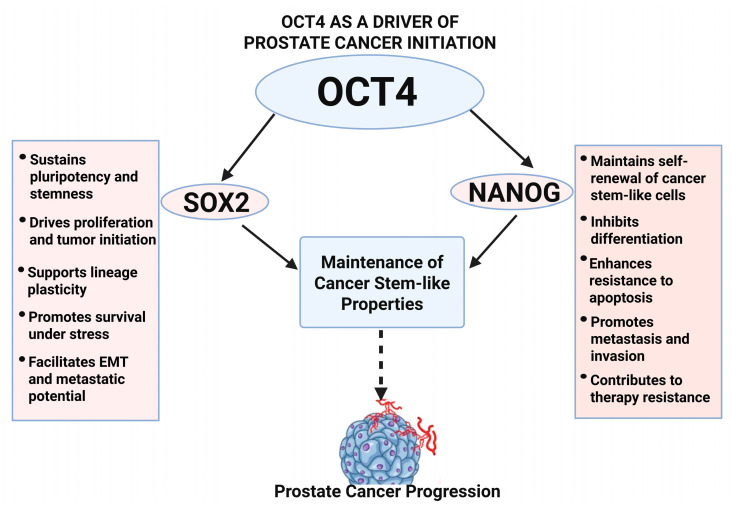
OCT4-mediated network in prostate cancer initiation. This diagram shows how OCT4 collaborates with SOX2 and NANOG to maintain cancer stem-like properties, block differentiation, and drives prostate cancer initiation and early progression.

The role of OCT4 in PCa initiation is closely tied to its ability to maintain cancer stem-like properties. As a key pluripotency factor, OCT4 works with SOX2 and NANOG to sustain an undifferentiated, stem-like state, preventing normal differentiation processes and driving tumor progression (Figure 2). The persistence of cancer stem-like cells (CSCs) in PCa is a significant contributor to tumor heterogeneity, therapeutic resistance, and disease recurrence.

Multiple studies, like the one by Roy et al. [108], have established a strong association between OCT4 expression and aggressive PCa phenotypes. Its presence has been detected in prostate hyperplasia and malignant tissues, indicating its potential role as a cancer stem cell marker in PCa [109]. High OCT4 expression has been linked to increased tumorigenicity in prostatic tissues, supporting its role in disease initiation and progression [41].

Moreover, OCT4 is upregulated via Nodal, a member of the TGF-β signaling family, further reinforcing its function in sustaining a stem-like state in PCa cells. Activating this pathway promotes a self-renewing and undifferentiated phenotype, contributing to the aggressiveness of the disease [110].

### 3.2. Association of OCT4 with Prostate Cancer Progression and Metastasis

The impact of OCT4 on the PCa progression extends beyond its role in tumor initiation. Elevated OCT4 expression is strongly associated with several clinical and pathological features that define aggressive PCa, including the following:

- Increased Tumor Grade and High Gleason Score

OCT4 isoforms demonstrate divergent associations with the prostate cancer prognosis. The OCT4A expression correlates with higher Gleason scores, increased proliferation, and reduced differentiation—features of a more aggressive tumor state. In contrast, OCT4B expression is linked to lower Gleason scores and improved biochemical recurrence-free survival, suggesting a potential role as a favorable prognostic biomarker [111].

- Enhanced Metastatic Potential

One of the most critical aspects of PCa progression is metastasis. OCT4 has been directly linked to increased motility, invasiveness, and metastatic spread. Studies have demonstrated the following:

OCT4+ CSCs are associated with visceral metastases, suggesting that cells expressing OCT4 have a greater ability to invade distant organs [22]. Nong et al. [112] reported that the OCT4 expression and OCT4-related polymorphisms were associated with a larger tumor size, lymph node involvement, and distant metastases, supporting a potential role for OCT4 in cancer dissemination and progression. OCT4 has been identified as a key driver of metastasis in a Galectin-dependent manner, indicating a novel regulatory mechanism through which it promotes PCa cell migration and invasion [113].

- Poor Patient Prognosis

Clinical studies have shown that patients with higher OCT4 expression levels tend to have poorer survival outcomes. Increased OCT4 expression correlates with the following:

- Higher recurrence rates following treatment and shortened overall survival in patients with advanced PCa [114].
- Resistance to conventional therapies, including androgen ADT, chemotherapy, and targeted therapies [115,116].

### 3.3. OCT4 and Therapy-Induced Lineage Plasticity in Prostate Cancer

A growing body of evidence suggests that androgen receptor (AR)-targeted therapies not only fail to fully eliminate prostate cancer (PCa) cells but may also promote lineage plasticity, contributing to the development of castration-resistant prostate cancer (CRPC)—a form of the disease that continues to progress despite androgen deprivation therapy [117]. This therapy-induced plasticity plays a pivotal role in treatment resistance and disease progression. Over time, some CRPC tumors may undergo further transdifferentiation, resulting in the emergence of neuroendocrine prostate cancer (NEPC), a highly aggressive and AR-independent subtype characterized by neuroendocrine features and poor prognosis [118,119,120].

OCT4, a key regulator of stemness and cellular plasticity, has been implicated in driving therapy-induced lineage transition in prostate cancer. Nuclear Oct4A expression has been observed in neuroendocrine prostate cancer cells [121], and pluripotency factors including OCT4 are upregulated during AR-negative/CRPC lineage reprogramming [15].

#### 3.3.1. The Role of AR-Targeted Therapy in Driving Stemness and Plasticity

ADT- and AR-signaling inhibitors (ARSIs) like enzalutamide and abiraterone are standard treatments for advanced PCa [122,123]. While these therapies are initially effective in suppressing tumor growth, PCa cells often develop resistance by activating alternative survival pathways. One of the most well-documented responses to AR-targeted therapy is the upregulation of pluripotency-associated transcription factors, including OCT4, SOX2, and NANOG, which promote a stem-like transcriptional program [26,124,125].

PCSCs (Prostate Cancer Stem Cells) are largely AR-negative, allowing them to evade androgen deprivation therapy and sustain tumor growth through alternative survival pathways [126]. OCT4 enhances cellular plasticity by blocking differentiation pathways and maintaining a dedifferentiated, therapy-resistant state [127]. Also, AR-targeted therapies, particularly enzalutamide, have been shown to drive PCa stemness and lineage plasticity by enriching a therapy-resistant, stem-like-cell population [128,129]. Gene network and pathway analyses have identified OCT4, SOX2, and NANOG as key regulators of this process, promoting self-renewal and drug resistance through pluripotency-associated signaling pathways. Elevated OCT4 expression in enzalutamide-resistant cells reinforces its role in sustaining cancer stemness and driving therapy-induced lineage plasticity [130]. Additionally, CSC-enriched populations exhibit heightened resistance to therapy, further strengthening the role of OCT4 in sustaining cancer stemness [131]. The identification of OCT4-driven plasticity in enzalutamide-resistant cells highlights its role as a key mediator of therapy-induced lineage plasticity, making it a promising target for overcoming treatment resistance in PCa [36]. The interplay between cellular plasticity and stemness factors like OCT4, SOX2, and NANOG enables PCa cells to adapt under therapeutic pressure, contributing to tumor heterogeneity, EMT activation, and drug resistance [132]. In the context of NEPC, a research group suggests that OCT4A-expressing cells are increasingly detected in high-grade PCa and are associated with neuroendocrine differentiation, as they co-express chromogranin A and synaptophysin [121]. Formaggio N et al. [25] mentioned that OCT4 contributes to the dedifferentiation of PCa into an AR-negative state, promoting neuroendocrine differentiation and reducing sensitivity to AR-targeted therapies. Its expression is elevated in cells with RB1 and TP53 loss, though it alone may not be sufficient to drive enzalutamide resistance [133]. Recently, Kainulainen et al. [134] found that the inflammatory tumor microenvironment (TME), particularly tumor-associated macrophages (TAMs), significantly influences PCa plasticity. Pro-inflammatory M1 macrophages secrete factors that promote stemness by upregulating OCT4, SOX2, NANOG, KLF4, and CD44, while simultaneously suppressing AR signaling in PCa cells [134]. Additionally, OCT4 has been implicated in mediating resistance to chemotherapeutic agents like docetaxel and mitoxantrone, with drug-resistant PCa cells exhibiting increased OCT4 expression and tumor-initiating capacity. This resistance may arise through epigenetic reprogramming, including demethylation of the POU5F1/OCT4 locus, or the selection of pre-existing OCT4-positive subpopulations [135].

#### 3.3.2. Chromatin Modifications in Driving Stemness and Plasticity in Prostate Cancer Contributing to Drug Resistance

PCa is highly sensitive to epigenetic regulation, which contributes significantly to tumor progression, therapy resistance, and lineage plasticity. A study by Chen et al. [136] revealed that oncogenes in PCa can acquire super-enhancers (SEs), leading to transcriptional addiction and enhanced tumorigenicity. These SEs regulate key pluripotency genes, such as OCT4, SOX2, and NANOG, thereby maintaining a stem-like cancer cell state.

OCT4, in particular, exerts epigenetic control through multiple mechanisms. AlAbdi et al. [137] demonstrated that OCT4 inhibits Lsd1 activity, preventing H3K4me1 demethylation and DNA methylation at developmental enhancers, creating a “primed” enhancer landscape. This state preserves chromatin accessibility for oncogenic transcription factors, thereby fueling tumorigenesis, metastasis, and resistance to therapy. Furthermore, OCT4 interacts with chromatin modifiers and transcriptional networks to block differentiation and sustain a dedifferentiated, stem-like phenotype, with its enhancer-promoter interactions and chromatin remodeling activities directly contributing to drug resistance [138]. Epigenetic modifications, particularly histone methylation and acetylation, also regulate the expression of pluripotency factors. For instance, active histone marks drive SOX2 and OCT4 transcription in PCa. Treatments with epigenetic drugs such as AZA, TSA, and DZNeP can reverse these chromatin changes, reduce OCT4 expression, and impair cancer cell viability [139].

In research published in Nature Cell Biology, the authors demonstrated that EZH2 plays a central role in driving lineage plasticity in prostate cancer, promoting neuroendocrine trans differentiation following androgen receptor (AR) inhibition. Within this framework, OCT4 was identified as a downstream effector contributing to cellular reprogramming. OCT4^+^ cells, tracked using a dual-reporter system, acquired neuronal-like traits and co-expressed ASCL1. Transcriptomic analysis revealed that these cells were enriched for genes involved in plasticity and epigenetic regulation, with enhanced EZH2 activity observed in the OCT4^+^ population [140]. Additionally, OCT4 plays a key role in transcription factor (TF) network formation in CRPC and NEPC by interacting with FOXA1/AR in AR-positive PCa and NRF1 in AR-negative cases. OCT4 occupies super-enhancer (SE) regions, where it helps organize transcription factor hubs and facilitates phase-separated transcriptional condensates—a process that amplifies oncogenic transcriptional output [15].

Beyond AR-targeted therapy resistance, chromatin remodeling plays a crucial role in PCa lineage plasticity, acting as an epigenetic “memory” that allows cells to transition between different states. A recent study by Logotheti et al. [141] identified distinct subtypes of CRPC with unique chromatin and transcriptional landscapes, including an OCT4-associated stem-like (CRPC-SCL) subtype defined by high CD44 expression. This suggests that OCT4-driven plasticity extends beyond AR-signaling suppression, contributing to broader dedifferentiation processes. In two studies by the same research group, MUC1-C was shown to promote lineage plasticity in NEPC by activating E2F1, which induces the PBAF chromatin remodeling complex, and by driving the MYC→BRN2→SOX2 pathway. These mechanisms collectively lead to the induction of pluripotency-associated transcription factors—including OCT4, SOX2, KLF4, and MYC—thereby reinforcing stemness and plasticity in prostate cancer [87,142]. Shokraii et al. [143] explained that in PCa stem-like cells (OCT4, SOX2, NANOG positive), epigenetic modifications, particularly histone alterations like increased H3K27me3 and decreased H3K4me3, drive CDH1 repression, enhancing metastasis. Saha et al. [144] claim that OCT4 plays a key role in PCa epigenetic regulation by influencing the stemness, lineage plasticity, and tumor progression. They mentioned that its variant, OCT4A, is crucial for self-renewal, while OCT4 pseudogenes (POU5F1P1, POU5F1P3, POU5F1P4) contribute to cancer development.

Epigenetic mechanisms, including histone modifications and DNA methylation, regulate OCT4 expression, affecting therapy resistance and clinical outcomes [137]. Moreover, epigenetic editing using dCas9 fusion proteins has demonstrated the ability to regulate gene expression in PCa. Notably, dCas9-CBP has been shown to upregulate OCT4 expression, highlighting its role in epigenetic reprogramming [145]. Transposable elements (TEs) also serve as epigenetically regulated hubs for transcription factors binding during fate transitions. In PCa, TEs are co-opted to support oncogenesis, shifting from pluripotency transcription factors like OCT4 to lineage-specific factors such as AR. Despite similarities with pluripotent stem cells, PCa cells hijack TE regulation to favor tumor progression. Epigenetic modifications at TEs, including chromatin state alterations, play a crucial role in this process [146].

### 3.4. OCT4 as a Therapeutic Target in Prostate Cancer

Androgen deprivation therapy promotes the expansion of cancer stem cells (CSCs) by altering the tumor microenvironment and upregulating key stem cell markers, including OCT4, SOX2, NANOG, CD133, and CD44, driving tumor initiation, progression, and recurrence in PCa [147,148]. While various therapeutic approaches targeting CSCs—such as small-molecule inhibitors, monoclonal antibodies, and combination therapies—have shown promise, long-term resistance remains a major challenge, emphasizing the need for improved treatment strategies. Inhibitors of signaling pathways such as Hedgehog, Wnt, Notch, and PI3K/AKT have shown potential, and targeting the CSC microenvironment—via VEGF or CXCR4 signaling—may further disrupt CSC maintenance [149]. Immunotherapy strategies, including CAR-T cells and dendritic cell vaccines targeting CSC markers like CD44, EpCAM, and CD133, represent emerging approaches to overcoming resistance and improving treatment outcomes [150]. However, no single approach has been fully effective, underscoring the importance of combination therapies that target both CSCs and bulk tumor cells to enhance therapeutic efficacy in PCa.

Given its critical role in tumor progression, therapy resistance, and metastatic potential, OCT4 has emerged as a compelling therapeutic target in PCa [15,151]. Strategies to inhibit OCT4 expression or disrupt its downstream signaling pathways could provide novel treatment avenues, particularly in CRPC and NEPC, where current treatment options remain limited [119,152,153]. Recent studies have highlighted the role of the tumor microenvironment in regulating OCT4 expression. For instance, Kainulainen et al. [134] demonstrated that pro-inflammatory M1 macrophages enhance PCa cell plasticity by secreting factors that upregulate OCT4, NANOG, SOX2, KLF4, and CD44 via NFκB activation while suppressing AR signaling. Inhibition of NFκB signaling using the IKK16 inhibitor reversed this effect, reducing CSC marker expression and plasticity. MicroRNAs (miRNAs) have also been identified as key regulators of PCSCs. miR-100, miR-143, and miR-145 suppress PCa stemness by targeting essential transcription factors, including OCT4, KLF4, and c-Myc. Specifically, miR-100 downregulates AGO2, which influences stemness, while miR-143 and miR-145 directly inhibit OCT4 and other factors, thereby reducing tumorigenesis, migration, and invasion in PCa cells [154].

Natural compounds have shown promise in targeting PCSCs. Capsaicin treatment was found to reduce the expression of key PCSC markers, including CD44, CD133, ALDH1A1, SOX2, OCT4, and NANOG, in PC-3 and DU145 cells. This suppression of CSC traits was associated with the inhibition of the Wnt/β-catenin signaling pathway, ultimately leading to reduced cell growth [155]. Hormonal therapy has also been linked to CSC regulation. Guo et al. [29] found that enzalutamide modulates CSC marker expression by regulating SALL4, which influences OCT4 and SOX2 expression, thereby contributing to PCa progression. Targeting the AR/SALL4/OCT4-SOX2 pathway using sh-SALL4 may help mitigate enzalutamide-induced CSC expansion and invasion [29]. Similarly, TR4 has promoted chemoresistance to docetaxel in PCa by suppressing miR-145 expression, leading to increased OCT4 levels and enhanced resistance. Restoring miR-145 expression has been suggested as a potential strategy to counteract TR4-mediated chemoresistance [156,157]. Moreover, Fibroblast growth factor receptor 1 (FGFR1) has been identified as a crucial regulator of PCSCs. A study by Juyeon Ko et al. [28] demonstrated that OCT4-enriched 3D spheroids rely on FGFR signaling for survival. Inhibition of FGFR with BGJ398 or Dovitinib significantly reduced CSC proliferation, suggesting that targeting FGFR signaling could be a promising strategy for treating AR-independent CRPC.

Efforts to directly target OCT4-driven CSCs have led to innovative therapeutic strategies. Vaddi et al. [36] developed a SORE6-driven thymidine kinase (TK) suicide gene system designed to selectively eliminate SOX2/OCT4-overexpressing PCSCs. This system effectively blocked tumor progression and increased chemotherapy sensitivity, highlighting its potential as a therapeutic approach. Additionally, a study identified OCT4 as a key transcription factor in CRPC and NEPC, collaborating with FOXA1, AR, and NRF1 to drive the tumor progression. The antiviral agent Ribavirin was shown to disrupt these transcription factor interactions, sensitizing PCa cells to chemotherapy and slowing disease progression [15].

**Figure 3:**
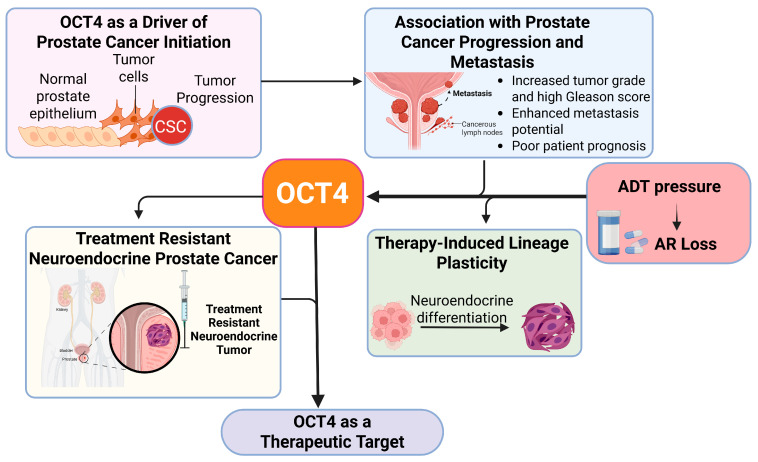
Role of OCT4 in prostate cancer progression and lineage plasticity. This figure shows how OCT4 helps prostate cancer cells change their identity, allowing the tumor to grow and become resistant to treatment. OCT4 promotes cell plasticity, which means cancer cells can switch to different types that survive even when hormone therapy is used.

Together, these findings highlight the pivotal role of OCT4 in maintaining CSCs, promoting therapy resistance, and facilitating disease progression in PCa. Targeting OCT4 and its associated pathways, either directly or through combinatorial strategies, holds great potential for improving treatment outcomes in advanced PCa (Figure 3).

## 4. Glimpse into the Future

**Figure 4:**
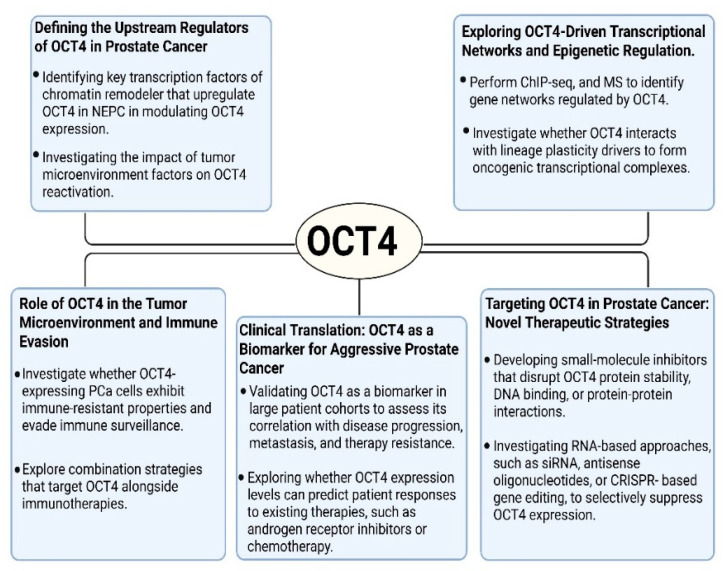
Future research directions for understanding and targeting OCT4 in prostate cancer. These schematics highlight five major areas of ongoing investigation: upstream regulators of OCT4 expression, its transcriptional and epigenetic networks, roles in immune evasion and the tumor microenvironment, potential as a clinical biomarker, and therapeutic strategies aimed at targeting OCT4 or its regulatory pathways.

Significant progress has been made in understanding OCT4′s role in PCa, but many questions remain unanswered, presenting new avenues for future research. Given its critical involvement in lineage plasticity, therapy resistance, and the transition to NEPC, further investigation into OCT4′s molecular regulation and therapeutic targeting is essential. Below, we outline several promising directions for future research on OCT4 in PCa (Figure 4).

*1.*  *Defining the Upstream Regulators of OCT4 in Prostate Cancer*

Although OCT4 is well-established as a master regulator of pluripotency, its reactivation in PCa is still not fully understood. Investigating the signaling pathways and transcriptional regulators that drive OCT4 expression in PCa, particularly in response to ADT or other treatment pressures, could uncover novel mechanisms of therapy resistance. Potential areas of study include [158] the following:

- Identifying key transcription factors or chromatin remodelers that upregulate OCT4 in CRPC and NEPC.
- Investigating the role of long non-coding RNAs (lncRNAs) and microRNAs (miRNAs) in modulating the OCT4 expression.
- Investigating the impact of tumor microenvironment factors, including hypoxia, inflammatory cytokines, and stromal interactions, on the OCT4 reactivation.

*2.*  *Exploring OCT4-Driven Transcriptional Networks and Epigenetic Regulation*

OCT4 functions as a transcription factor, orchestrating gene expression programs that maintain stemness and promote plasticity. However, its downstream targets and transcriptional co-factors in PCa remain largely unknown [159]. Future studies should

- Perform chromatin immunoprecipitation sequencing (ChIP-seq), RNA sequencing (RNA-seq), and mass spectrometry (MS) to identify the gene networks regulated by OCT4 in PCa.
- Investigate whether OCT4 interacts with other lineage plasticity drivers, such as SOX2, NANOG, EZH2, or AURKA, to form oncogenic transcriptional complexes.
- Examine the role of super-enhancers (SEs) in sustaining OCT4 expression and whether disrupting SEs could be a viable therapeutic approach.

*3.*  *Role of OCT4 in the Tumor Microenvironment and Immune Evasion*

Emerging evidence suggests that cancer stem-like cells may contribute to immune evasion. These kinds of studies make the research field more clinically relevant because the tumor microenvironment and the immune system are included [160]. Future studies should

- Investigate whether OCT4-expressing PCa cells exhibit immune-resistant properties and evade immune surveillance.
- Determine whether OCT4 influences immune checkpoint expression (e.g., PD-L1) or modulates tumor-associated macrophages, myeloid-derived suppressor cells (MDSCs), or T cells in the tumor microenvironment.
- Explore combination strategies that target OCT4 and other key master regulators—such as SOX2, MYC, EZH2, and BRN2—alongside immunotherapies, to more effectively disrupt the stemness and immune-evasive phenotypes associated with lineage plasticity in prostate cancer.

*4.*  *Clinical Translation: OCT4 as a Biomarker for Aggressive Prostate Cancer*

The clinical relevance of OCT4 in PCa, particularly in advanced subtypes such as CRPC and NEPC, is increasingly recognized but remains underexplored. Recent technological advances in liquid biopsy—including the analysis of circulating tumor cells (CTCs) and cell-free nucleic acids—offer promising non-invasive tools for tracking tumor evolution and treatment response [161]. Furthermore, elevated OCT4 expression has been linked to primary resistance to AR inhibitors, including enzalutamide and abiraterone. In patients with high pre-treatment OCT4 levels, poor response rates and early relapse have been reported. These findings support the potential of OCT4 as a predictive biomarker that could inform patient stratification and the selection of combination therapies aimed at delaying or circumventing resistance [10,147]. Future research should focus on the following:

- Validating OCT4 as a biomarker in large patient cohorts to assess its correlation with disease progression, metastasis, and therapy resistance.
- Developing non-invasive diagnostic tools (e.g., CTCs, exosomal OCT4 detection) to monitor disease progression in CRPC and NEPC patients.
- Exploring whether OCT4 expression levels can predict patient responses to existing therapies, such as androgen receptor inhibitors or chemotherapy.

*5.*  *Targeting OCT4 in Prostate Cancer: Novel Therapeutic Strategies*

Given its role in therapy resistance, OCT4 represents an attractive target for drug development. However, direct inhibition of transcription factors remains challenging, and there is a lack in this area, especially in the clinic [162]. Future therapeutic strategies should explore the following:

- Developing small-molecule inhibitors that disrupt OCT4 protein stability, DNA binding, or protein–protein interactions.
- Investigating RNA-based approaches, such as siRNA, antisense oligonucleotides, or CRISPR-based gene editing, to selectively suppress OCT4 expression.
- Identifying upstream regulatory pathways (e.g., NFκB, FGFR, or Wnt/β-catenin) that indirectly modulate OCT4 and could be targeted with existing inhibitors.
- Exploring the potential of targeted protein degradation strategies, such as PROTACs (proteolysis-targeting chimeras), to selectively degrade OCT4 in PCa cells.

## 5. Conclusions

OCT4 plays a central role in the prostate cancer (PCa) progression by regulating stem-ness, plasticity, and resistance to therapy. Its activity is modulated by multiple oncogenic pathways, including Wnt/β-catenin, PI3K/AKT, STAT3, and NF-κB, as well as through chromatin remodeling and tumor microenvironmental cues [163,164,165,166].

In PCa, OCT4 emerges as a driver of lineage plasticity, therapy resistance, and the transition to aggressive subtypes such as CRPC and NEPC. By maintaining cancer stem cell populations and promoting epithelial–mesenchymal transition (EMT), OCT4 enables tumor cells to evade standard therapies and acquire more invasive and treatment-resistant characteristics. Its ability to suppress AR signaling and facilitate androgen-independent growth further underscores its significance in PCa pathophysiology. Given its broad impact on tumor evolution, OCT4 represents a compelling target for therapeutic intervention.

Given OCT4′s intracellular localization and lack of enzymatic activity, directly targeting this transcription factor remains challenging. However, progress in developing RNA-based interventions (e.g., miRNAs, siRNA), CRISPR/dCas9 systems, and epigenetic therapies has opened new possibilities. These strategies either suppress the OCT4 expression or inhibit its downstream effects. Additionally, targeting regulators or microenvironmental factors that indirectly modulate OCT4—such as inflammatory macrophages or upstream oncogenic signals—offers another route to mitigate its pro-tumor functions. Combination therapies that integrate the targeting of OCT4 and other master regulators—such as SOX2, MYC, and EZH2—with standard treatments like ADT, chemotherapy, or immunotherapy may provide a more comprehensive strategy for overcoming resistance and improving patient outcomes.

Beyond its therapeutic potential, OCT4 also holds promise as a prognostic and predictive biomarker in PCa. Elevated OCT4 expression has been associated with poor clinical outcomes, including increased metastatic potential and reduced overall survival. Therefore, incorporating OCT4 assessment into clinical decision-making could help identify patients at a high risk of aggressive disease progression and therapy failure. Future studies should focus on validating OCT4 as a biomarker in large patient cohorts and exploring its utility in guiding treatment selection [22].

Ultimately, while targeting OCT4 and other stemness factors presents challenges, their central role in PCa lineage plasticity and therapy resistance makes it a key focus for future research. A deeper understanding of the molecular mechanisms governing stemness factor’s function, as well as the development of more effective therapeutic strategies, could pave the way for novel treatment approaches aimed at overcoming resistance and improving survival outcomes for patients with advanced PCa.

## Figures and Tables

**Table 1:** Key signaling pathways regulating OCT4 in CSCs.

Signaling Pathways	Key Role in Lineage Plasticity	Interaction with OCT4	Downstream Effector	Cancer Type	Therapeutic Implication	Clinical Evidence Level	References
**Wnt/β-catenin**	Elevation in CSC-like properties, promotes dedifferentiation, induces transcription, and enhances epithelial–mesenchymal transition, Angiogenesis, and Antiapoptotic effect, drug resistance	Promote the promoter activity of OCT4 and upregulate its expression. OCT4 overexpression increased the WNT signaling pathway. OCT4 knockdown decreased Wnt/β-catenin pathway downstream genes. OCT4 Activation Expressed together Forms complex with OCT4 and enhances its activity Forms complex with OCT4 and modulates its activity Activation of CSC marker, including OCT4 OCT4 Activation β-catenin stabilizes OCT4 expression in CSCs, and OCT4 is a critical mediator of CSC stemness in this pathway	CagA (Cytotoxin-associated gene A). LEF1 (lymphoid enhancer binding factor 1). Wnt10b Ang1, bFGF, HGF, VEGF, Bcl2 Wnt1 Nanog, Tbx3, Tcfcp2L1, and PDGFα β-catenin, TAMs (Tumor-Associated Macrophages), IL6, OV6+ CSCs.	Human Gastric Cancer Hepatocellular Carcinoma PBMSCs ESCs mESC Human Ovarian Cancer Breast Cancer PCa	Wnt/β-catenin inhibitor (xav939) Curcumin, Wnt/β-catenin pathway inhibitor GSK-3 inhibitor (CHIR99021) CCL16 inhibition (GSK3β or STAT3-targeted inhibitor) Targeting both CSCs and TAMs, using IL6R inhibitor (tocilizumab).	Preclinical (XAV939, CHIR99021, CCL16 inhibition). Clinical (Tocilizumab). Preclinical/Clinical (Curcumin)	[72,73,74,75,76,77,78,79,80]
**PI3K/AKT/mTOR**	Enhance proliferation, EMT, and plasticity	AKT phosphorylates OCT4. Knockdown OCT4 downregulates the expression of p-PI3K, p-AKT, and p-mTOR. AKT phosphorylates OCT4 and increases its stability and nuclear localization. Reciprocal regulation with OCT4 (negative and positive feedback). Induces OCT4 and SOX2 expression through WAVE3 signaling. Promotes OCT4 and SOX2 expression.	p-OCT4 p-PI3K, p-AKT, and p-mTOR p-OCT4, AKT1, SOX2, NANOG mTOR CA125, WAVE3 CD44, MMP-2, MMP-9	Glioblastoma Liver cancer Ovarian Cancer Embryonal Carcinoma Ovarian Cancer Nasopharyngeal Carcinoma (NPC)	Inhibition of AKT (Akti-1/2) Metformin (Inhibition of OCT4 Activity). Inhibition of AKT (via LY294002). mTOR signaling pathway inhibition (rapamycin)	Preclinical (Akti-1/2, LY294002). Clinical (Metformin, Rapamycin)	[23,81,82,83,84,85]
**TGF-β**	Induces EMT and plasticity	OCT4 knockdown decreased TGF-β pathway downstream genes. OCT4 could induce TGFBR2 transcription. Expressed together	ELF, Smad3, Smad4 T-reg-like effector genes Smad3 and Smad4	Hepatocellular Carcinoma GBM	inhibition of TGFBR2	Preclinical	[74,76,86]
**Notch1**	Drives CSC maintenance, tumor aggressiveness promotes therapy resistance, and integrates EMT with CSC self-renewal	Interacts with OCT4 and NANOG to expand CSC populations. NOTCH1 is upregulated in EMT-like cells and contributes to stemness by increasing OCT4, NANOG, and SOX2. NOTCH1 overexpression correlates with increased OCT4, NANOG, and SOX2, promoting cancer stem-like cell (CSC) features Notch1 and OCT4 expression levels are positively associated. miR-221/222 downregulates Reck and activates OCT4 expression through Notch1 signaling. EGFR inhibition increases CSC-like properties via Notch1 activation, upregulating OCT4 and Bmi-1 Notch1 activation correlates with increased expression of stemness markers like OCT4 STC1 secreted by CAFs activates Notch1 signaling in HCC cells, promoting stemness YAP1-Notch1 positive feedback loop enhances stem cell marker expression such as OCT4	HES1, HEY1, NANOG. Lin28B, miR-200, let-7, Polycomb Repressor Complex 2 (EZH2, SUZ12, EED), ZEB1, Vimentin Snail, Slug, ZEB1/2, Vimentin (↑), E-cadherin (↓) CD133 NICD HES1, Bmi-1 Hes1, Hey1, Jagged1 Hes1, Hey1, Cyclin D1, c-Myc BMP4-SMAD1/5	Prostate Cancer Gastric Carcinoma NSCLC Salivary Adenoid Cystic Carcinoma Hepatocellular Carcinoma Breast Cancer	MUC1-C or Notch1 inhibition. Natural agents to downregulate miR-200. GSI inhibitor (Notch inhibition) Combined targeted therapy Targeting miR-221/222 γ-secretase inhibitor GSI to downregulate Notch1 Signaling Notch1 inhibition using LY3039478 (a novel r-secretase inhibitor) Suggesting inhibition of STC1 and/or Notch1 Suggesting Targeting Notch1 signaling in combination with YAP1 and BMP4 pathways	Preclinical (MUC1-C, miRNAs, STC1, YAP1/BMP4). Clinical trials (GSI, LY3039478 in non-PCa tumors).	[87,88,89,90,91,92,93,94,95]
**JAK1-STAT3**	Promotes CSC plasticity and enhances tumor progression, viability, migration, and invasion. Contributes to chemoresistance and poor prognosis.	OCT4 overexpression in HCC is correlated with increased survivin expression and STAT3 phosphorylation, indicating activation of the JAK/STAT pathway. IL-6 activates JAK1/STAT3 signaling, leading to OCT4 upregulation and conversion of non-CSCs to CSCs. Dox-induced Stat3 activation upregulates OCT4 and c-Myc, promoting CSC enrichment and drug resistance LPS and TNF-α increase OCT4 expression, activating invasion pathways through Stat3 signaling. MAPKi treatment activates STAT3, leading to the upregulation of OCT4 and SOX2, suppressing apoptosis and enhancing resistance to therapy. STAT3 directly upregulates OCT4 expression, maintaining CSC properties	Survivin IL6, VEGF, MMP9 c-Myc, CD44, and ABCG2 JNK1, p65, and STAT3 HIF-1α, Nanog, Sox2, CD44	hHepatocellular Carcinoma Breast Cancer TNBC (Triple Negative Breast Cancer OSCC (oral squamous cell carcinoma Melanoma Esophageal Adenocarcinoma Cells	OCT4 Silencing Stat3 inhibition via such as niclosamide and LLL12 or using anti-IL-6 antibody. Stat3 inhibitor WP1066 Alantolactone, a STAT3 inhibitor, combined with MAPKi.	Preclinical (OCT4 silencing, LLL12, alantolactone). Clinical trials (niclosamide, WP1066, anti-IL-6 antibody).	[96,97,98,99,100,101]
**ERK/MAPK**	Regulates tumor progression, differentiation, apoptosis, and EMT. Promotes CSC-like properties	Co-activated with OCT4, SOX2, Myc, and Notch Bach1 interacts with OCT4, Sox2, and Nanog to maintain CSC identity. OCT4, NANOG, and SOX2 are upregulated in paclitaxel-resistant LUAD cells (A549/TAX) alongside MUC1, contributing to CSC maintenance and therapy resistance. MK2 phosphorylates OCT4, enhancing its transcriptional activation of c-MYC and promoting tumor progression and therapy resistance. FL3 activates p38 MAPK, leading to caspase-3-mediated apoptosis and suppression of OCT4 expression in cancer stem-like cells.	E2F, Myc, SOX-2, TGF-β, OCT4 CD44 EGFR, IL-6, CD133 c-MYC, MYCN, pMK2 Caspase-3	Gastric Cancer Lung Cancer LUAD (Lung Adenocarcinoma) Neuroblastoma Teratocarcinoma	ERK/MAPK Kinase inhibitor PD98059 MAPK inhibitor (LY2228820). Suggest targeting MUC1 and MAPK pathways in combination with paclitaxel. MK2 inhibition (PF3644022, MK2iIII). MAPK inhibition by SB203580.	Preclinical (PD98059, SB203580, MK2 inhibitors, MUC1 combo). Clinical trials (LY2228820/ralimetinib)	[102,103,104,105,106]
